# A Perspective for Clinical Pharmacy Curriculum Development and Validation in Asian Developing Nations

**DOI:** 10.4103/0975-1483.80304

**Published:** 2011

**Authors:** TM Khan, M Anwar, KK Mueen Ahmed

**Affiliations:** *College of Clinical Pharmacy, King Faisal University, Al-Ahsa, Saudi Arabia*; 1 *School of Pharmacy, University of Otago, NewZealand*

**Keywords:** Pharmacy curriculum, asian developing nations, curriculum development, validation

## Abstract

This perspective is a reflection of the Personal teaching experience of the authors. The aim of this perspective is to identify the weaknesses in the pharmacy curriculum development in Asian developing nations and to propose a methodological approach for curriculum validation. It has been seen that improper selection of the course contents were the common limitations found in the pharmacy curriculum in developing nations. Furthermore, lack of facilities and improper student evaluation system were the other flaws that are acting as a main challenge to pharmacy education in developing nations. A systemic way for the curriculum designing and validation can be a solution to manage the observed deficiencies. Keeping in view this motivation a set of task are defined in the form of Pharmacy Curriculum Development and Validation Model (PCDVM) that can be a guideline for the pharmacy educators for the evaluation and validation of the curriculum. Partial or full implementation of this model will enable the pharmacy institutions to deliver quality knowledge to pharmacy students which will further contribute to quality health care system in developing countries.

## INTRODUCTION

Curriculum development in the context of pharmacy education is a critical issue under discussion from the last decade.[1] Advancements in the pharmaceutical sciences and progressing role of pharmacist in direct patient care are forcing the pharmacy institutions to come up with new courses and modules to equip the future pharmacists with the updated knowledge in order to diversify their skills for better patient management.[2] However, it is seen that while designing and reviewing the curriculum, certain aspects are often missed or neglected. This situation can easily be seen in the South Asian and Southeast Asian countries where pharmacy programs are in the trail phase from the last decade.[3,4] Currently, pharmacy education in South Asian and Southeast Asian countries is in transition phase with the upgradation of Bachelor of Pharmacy (B. Pharm.) to Doctor of Pharmacy (Pharm-D). Still it seems controversial why there was a need for this program; either it was based on true needs or it was just started to fulfil the needs of international standards. As the clinical pharmacy is at grass-root level in these countries, availability of skilled professionals is another issue that is a big question mark on the Pharm-D programs taught in South Asian and Southeast Asian countries. However, still many are happy with the Bachelor of Pharmacy (B. Pharm.), but dual education standards can be seen where universities have memorandum of understanding with British Universities which are offering M. Pharm. degrees which are equivalent to B. Pharm.[2]

The aim of this perspective is to highlight such issues and present a model which may act as a baseline for the curriculum development, curriculum revision and curriculum validation for pharmacy institutions in order to provide quality education and useful knowledge to the students. Some of the limitations which we have observed are illustrated below.

## LIMITATIONS IN THE PROCESS OF CURRICULUM DEVELOPMENT

In this particular aspect, the qualification and experience of the people involved in the curriculum development counts a lot. Qualification here does not mean the academic scrolls of the person who is developing the curriculum. Qualification in this context means the experience and capability to design the best possible curriculum,[5] which fulfils the national needs at the first glance and then the regional needs at the second. Particularly in South Asian and Southeast Asian countries, there is a deficiency not only of the clinical pharmacists but also of an ideal clinical pharmacy setup in health care system.[2] In this regard, the online available curriculum of the American and Canadian universities and periodic updates by the Accreditation Council for Pharmacy Education (ACPE) and American College of Clinical Pharmacy (ACCP) are the main source of inspiration for many South Asian and Southeast Asian countries. Often it is seen that curriculum is updated/revised or some time straight away copied from these resources. This practice gives rise to many questions such as whether or not this course fulfils the national needs, whether there are enough facilities in the local institutions and health care setting, which allow the students to practice thoroughly what they have learned theoretically. In other words, one can learn and see from the pre-developed courses and modify them according to the local needs and available facilities.

### Selection of course contents

Another neglected issue in terms of curriculum is the selection of course contents and their compliance with learning objectives. From the experiences of a franchised B. Pharm. program in Malaysia, it was observed/shared by the fellow colleagues that the course contents are not in compliance with the learning objectives and individual lectures that comprise the course. Similarly, in some cases, the time distribution is not appropriate and one cannot finish the described contents in the course manual in that time frame.[2] This improper distribution of time has a major impact on the students’ learning because the demonstrator does not have sufficient time to discuss all the issues in detail and students remain deprived of some valuable information that can be helpful in terms of practice.

Similarly, the selection of the appropriate reference books in some way does affect the course contents. Some of the first degree students have shared a thought, “reference book for this particular subject is very difficult; I am using another book as reference that is simpler and easier.”

### Strategies to evaluate students

It is very essential to adopt an affective method that results in an unbiased evaluation of the students. It is seen that the course evaluation is conducted on the basis of the course work, i.e. midterm quizzes, assignments, projects and final exam. Normally, the course works evaluation carries 30–40% of the marks and 60–70% of the marks are for the final exam. The midterm quizzes and final exam are the sole strategies that evaluate the personal knowledge of the students. However, assignments and group projects may result in biased evaluation of the students. It is often spotted that students often cut and paste the assignments from the electronic resources. This problem can be countered if a handwritten assignment is requested for, but in the case of group assignments like research reports/projects, evaluation can be more accurate if a viva is conducted for every student who participated in that project because in most of the group assignments, three out of ten students work very hard while the rest get the benefit without any effort. Some institutions have adopted the way of oral and poster presentations as an additional tool to evaluate student knowledge about the research design. However, these presentations again are not sufficient to identify the members who have minimal participation in these research projects because those students having higher contribution in these projects come at front to demonstrate about the project and all the members of the whole group get equal marks. Individual viva for every student will be one of the fair ways to mark and evaluate the student’s contribution, knowledge and understanding about the research project. For the clinical oriented subjects, the clerkships and hospital attachments are the main strategies which are merely 10–15% of the total marks. Clerkships in clinical oriented pharmacy programs are the most important training techniques to develop the students’ learning skills and to evaluate them. It would be better if clerkships have separate or individual marks than the course work. In this way, one can affectively make an estimation about the clinical skills of the students; moreover, students also get the proper benefit of their practical skills.

## PRACTICAL IMPLEMENTATION OF COURSE CONTENTS

After the curriculum development, the most important aspect is the practical implementation of the curriculum. Most of the time, a group of five to six academicians design a set of lectures for a course or a curriculum. Theoretically it is perfect but the practical execution will be better assured if someone from the field is involved while deciding the contents of the course. In this way, a more practically implacable program can be designed with minimum practical limitations. This is one of the most common deficiencies seen in the clinical pharmacy programs in South Asian and Southeast Asian countries.[2,3] But in the case where there is availability of skilled clinical professionals, a field survey among the practicing clinical pharmacists will be more affective to take final comments on the construct of the course. Moreover, this will help the institution to know what facilities they have and what is required in the future. For example, if an institution among the developing Asian countries designs a course of gene medicine for pharmacy students, then not only the students but also the academic staff and the institution will face a challenge in the practical implementation of the theoretical knowledge. Therefore, it will be more appropriate to keep in view the availability of the regional and national resources for any course that is designed or suggested to be a part of any pharmacy professional program.

Another neglected issue is the *pre-course students’ evaluation*, which is often missed by the majority. Pre-course evaluation is necessary to explore the student’s basic knowledge and the evaluation is also essential to find out what the students need to know in addition to the contents of the course. The best example is the subject of communication skill in pharmacy curriculum. The main aim of this subject is to develop good communication skills among the students and to further equip them with the techniques to counter the patient–pharmacist communication problems in the practice. But no one has ever evaluated how many of the students have apprehension in communicating with professional peers, friends, family members, etc. If communication skill is taught to a student with communication apprehension, then what skills he will develop if his/her basic problem is not solved. Ideal pedagogical approaches will be used to evaluate all the students for the possible prevalence of communication apprehension. Following this, a crash program will be offered to those having problem in communication apprehension. Then, instead of starting the course straight away with the first topic, there will be a brainstorming session that will discuss the entire course in detail with a brief glimpse of every lecture that is part of the course. Such problems are often faced by the newly enrolled students. Time and again, newly enrolled students ask questions such as “Why are we studying this particular subject,” “What is the need of this subject in pharmacy”, and “How do we prepare for the final exam”. Sometimes, the tutorials focusing on such questions are arranged in the study week. However, it will not be a wise choice to demonstrate the students about all these problems during the preparation week. If all this was done during the orientation week at the start of the semester, then it will be more fruitful for creating a better understanding for the students regarding their subjects. The final lecture should comprise the students’ queries and not the last topic of that particular course. Future studies on pedagogical approaches for pharmacy education can explore the nexus of mind mapping and brainstorming, with better understanding of the course content, scope and practical aspects.

## ASSESSMENT OF THE TEACHERS AND TEACHING METHODOLOGIES ADOPTED

Pharmacy teachers play a very vital role in establishing a good pharmacy practice setup in a country. They share quality information with their students, which will definitely be seen when they practice after graduation. But it is very difficult to define the criteria for a good pharmacy teacher. However, good communication skills and sound practical experience can be the criteria to define a good pharmacy teacher. Other aspects like healthy state of mind and teaching methodologies adopted act as a bonus.

So far, merely there is any committee or body that holds the powers to evaluate the teacher on the basis of knowledge and practical skill or very rarely someone has questioned the students about the didactics and practical style of teachers. The effort to evaluate the knowledge and educational skills is an essential need not only to delivery quality education to the students but also for designing an affective curriculum and later for the revision as well.[6] This limitation highlights the need to establish a committee at a national level, which is responsible for the teachers’ profile, not on sole basis, but the students should also be given an opportunity to share their views about the knowledge sharing efforts of teacher and pedagogical approaches adopted to share the knowledge. In addition, students should be given an opportunity to disclose about the communication skill of lecturers. Furthermore, it is seen that still many stick to the traditional classroom teaching approaches. Training of the teaching staff to adopt new teaching methods like problem based learning (PBL) will be an ideal way to further ease students’ understanding of the course contents.[2]

## RECOMMENDATION

**Figure 1:**
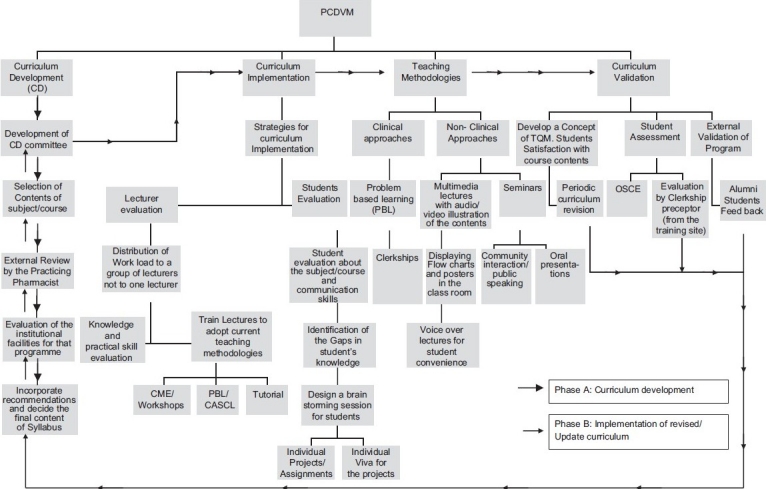
Pharmacy curriculum development and validation model

Keeping in view the limitations in the pharmacy education system in a developing nation, there is dire need of a model that not only provides grounds for affective curriculum development but also defines some way to validate the curriculum. Figure 1 can be a proposed baseline model that results in defining the ways not only for the development of an affective curriculum but also for its validation. Moreover, implementation of *Pharmacy Curriculum Development and Validation Model (PCDVM)* as a whole or with modifications will enable the pharmacy educator to produce pharmacists with good pharmacy skills that will be helpful in improving the direct patient care in the developing Asian nations.
